# Duration of red blood cell storage and outcomes in pediatric cardiac surgery: an association found for pump prime blood

**DOI:** 10.1186/cc8217

**Published:** 2009-12-21

**Authors:** Marco Ranucci, Concetta Carlucci, Giuseppe Isgrò, Alessandra Boncilli, Donatella De Benedetti, Teresa De la Torre, Simonetta Brozzi, Alessandro Frigiola

**Affiliations:** 1 Department of Cardiothoracic-vascular Anesthesia and Intensive Care, IRCCS Policlinico S. Donato, Via Morandi 30, San Donato Milanese, Milan, 20097, Italy; 2 Department of Cardiac Surgery, IRCCS Policlinico S. Donato, Via Morandi 30, San Donato Milanese, Milan, 20097, Italy

## Abstract

**Introduction:**

Cardiac surgery using cardiopulmonary bypass in newborns, infants and small children often requires intraoperative red blood cell transfusions to prime the circuit and oxygenator and to replace blood lost during surgery. The purpose of this study was to investigate the influence of red blood cell storage time prior to transfusion on postoperative morbidity in pediatric cardiac operations.

**Methods:**

One hundred ninety-two consecutive children aged five years or less who underwent cardiac operations using cardiopulmonary bypass and who received red blood cells for priming the cardiopulmonary bypass circuit comprised the blood-prime group. Forty-seven patients receiving red blood cell transfusions after cardiopulmonary bypass were separately analyzed. Patients in the blood-prime group were divided into two groups based on the duration of storage of the red blood cells they received. The newer blood group included patients who received only red blood cells stored for less than or equal to four days and the older blood group included patients who received red blood cells stored for more than four days.

**Results:**

Patients in the newer blood group had a significantly lower rate of pulmonary complications (3.5% versus 14.4%; *P *= 0.011) as well as a lower rate of acute renal failure (0.8% versus 5.2%; *P *= 0.154) than patients in the older blood group. Major complications (calculated as a composite score based on pulmonary, neurological, and gastroenterological complications, sepsis and acute renal failure) were found in 6.9% of the patients receiving newer blood and 17.1% of the patients receiving older blood (*P *= 0.027). After adjusting for other possible confounding variables, red blood cell storage time remained an independent predictor of major morbidity. The same association was not found for patients receiving red blood cell transfusions after cardiopulmonary bypass.

**Conclusions:**

The storage time of the red blood cells used for priming the cardiopulmonary bypass circuit in cardiac operations on newborns and young infants is an independent risk factor for major postoperative morbidity. Pulmonary complications, acute renal failure, and infections are the main complications associated with increased red blood cell storage time.

## Introduction

Cardiac surgery using cardiopulmonary bypass (CPB) in newborns, infants and small children requires the use of intraoperative homologous red blood cell (RBC) transfusions in the majority of cases. RBCs are used to prime the CPB circuit and oxygenator (although the most recently developed oxygenators require a very small priming volume) and to correct intraoperative anemia during and after CPB.

Allogeneic RBC transfusion has more of an impact on the physiology of pediatric patients than on adult physiology. During cardiac operations, patients weighing less than five kilograms may receive RBC transfusions that total more than 50% of their circulating blood volume, which is the equivalent of a massive (more than three liters) RBC transfusion in adults. It is well known that massive transfusions can be associated with a number of complications, both in critically ill adult patients and in adult patients undergoing cardiac surgery [1-3]. It is therefore reasonable to hypothesize that the same may happen in newborns, infants and small children undergoing cardiac surgery using CPB.

In a recent article, Koch and coworkers [4] elegantly demonstrated that the duration of RBC storage prior to transfusion was independently associated with increased morbidity and mortality in adult cardiac surgery patients as well as decreased long-term survival. This study confirmed the results of previous studies, which found an association between the risk of complications and blood storage time [5-7].

In this study, we tested the hypothesis that among newborns, infants and small children undergoing cardiac surgery using CPB, the storage time of the RBCs transfused during the operation may (i) cause changes in the metabolic profiles of the patients during CPB and (ii) lead to differences in postoperative complication rates. Postoperative transfusions in patients having undergone operations without blood prime or intraoperative transfusions were separately addressed in a sensitivity analysis.

## Materials and methods

This retrospective study enrolled 192 consecutive newborns, infants and small children who underwent cardiac surgery using CPB and who required RBC transfusion to prime the CPB circuit. A second group of 47 patients being transfused after CPB was separately analyzed. All patients underwent surgery at our institution between January 2006 and December 2008. The duration of RBC storage of the transfused blood was not available before January 2006 in our database. During the study period, 948 patients were operated on for congenital heart disease at our Institution. Two hundred forty-five were adult (>16 years) congenital patients, and 123 were excluded because they were operated on without CPB; the remaining 580 did not receive RBC transfusions to prime the CPB circuit: 98 of these patients received RBC transfusions after CPB, and the remaining were not transfused. It is our policy not to use blood prime in patients weighing more than 10 kg, unless they are severely anemic.

For patients needing a blood prime, it is our policy to ask the blood bank to provide us with RBCs stored for less than seven days; however, this is not mandatory, and depending on availability patients may receive RBCs stored for a longer period of time.

The study design was approved by the local Ethics Committee and the need for parental consent was waived given the retrospective nature of the study. The primary endpoint of the study was to determine patients' morbidity based on the duration of storage of the blood that patients received and to compare major morbidity rates in patients receiving newer vs. older blood. The secondary endpoint was to examine the metabolic profile of patients during CPB based on RBC storage time.

### Patients

Pediatric patients undergoing a cardiac operation using CPB during the study period in whom RBCs were used in the priming solution of the CPB circuit were included in the blood-prime group. The use of RBCs in the priming solution is a current practice at our institution in cases when the use of a crystalloid or colloid priming solution would result in a severe hemodilution. Patients receiving RBCs both in the priming solution and after CPB were included in this group. Patients receiving RBC transfusions only after CPB were separately analyzed (post-CPB transfusion group).

### Anesthesia, cardiopulmonary bypass, and cardiac surgery technique

Anesthesia was carried out according to our institutional practice. Induction of anesthesia was achieved with intravenous midazolam. A high-dose opioid anesthetic (fentanyl 50 μg/kg) was used for maintenance of anesthesia and supplemented with midazolam and sevoflurane as tolerated. Neuromuscular blockade was achieved with vecuronium. All patients underwent endotracheal intubation and were mechanically ventilated. Standard monitoring was used, which included a radial or femoral artery catheter for measurement of systemic arterial blood pressure and intermittent blood sampling, a double lumen right internal jugular or femoral central venous catheter, and esophageal and rectal temperature probes.

Cardiac cannulation was performed after intravenous administration of 300 IU/kg of unfractionated heparin and only after an activated clotting time of longer than 450 seconds was achieved. Additional heparin boluses were used to maintain an activated clotting time in this range before and during CPB. Double venous cannulation of the superior and inferior vena cava was generally performed. The arterial cannula was placed into the ascending aorta. The CPB circuit included a hollow fiber oxygenator (Dideco D901 or D902, Sorin Group, Mirandola, Italy) with an arterial line filter and a centrifugal pump (Bio-Medicus, Medtronic, Minneapolis, MN, USA).

In the blood-prime group the CPB circuit was primed with a solution containing RBCs and a 4% albumin solution. The solution was titrated to reach a hematocrit value of 30% once the patient was connected to the circuit and CPB was initiated. The total priming volume varied between 350 mL and 450 mL. Therefore, the amount of RBCs used in the priming solution varied according to the patient's baseline hematocrit, weight, and the priming volume used. In all patients, less than a 250 mL volume of RBCs and only one bag of stored RBCs were used for priming the circuit. Only one bag of stored RBCs was used to prime the circuit.

Patients in the post-CPB transfusion group received a 4% albumin solution for priming the CPB circuit. CPB flow was targeted at 150 mL/kg and subsequently adjusted according to the patient's temperature.

The target patient temperature was chosen by the surgeon based on the type of surgical procedure being performed and personal preferences. All procedures were performed using a regimen of mild (32°C to 34°C), moderate (26°C to 31°C), or deep (20°C to 25°C) hypothermia. Patients were treated with an alpha-stat strategy if mild hypothermia was used and with a pH-stat strategy if moderate or deep hypothermia was used.

Cardiac arrest was obtained and maintained using antegrade intermittent blood cardioplegia.

After completion of the CPB and removal of the cannulas, heparin was reversed using protamine sulfate at a 1:1 ratio.

During and after CPB, additional RBCs were administered as needed in order to maintain a hematocrit value within our standard range. These additional RBCs either came from the first blood bag or from a second blood bag. No patient received RBCs from more than two blood bags during the operation. No leukodepleted blood was used for intraoperative transfusions.

### Data collection

Pre- and intraoperative data were derived from our institutional database. Data collected included age (months), weight (kilograms), hematocrit (%), serum creatinine level (mg/dL), serum bilirubin level (mg/dL), platelet count (cells/μL), prothrombin activity (%), activated partial thromboplastin time (seconds), antithrombin activity (%), redo operations, type of operation, the Aristotle severity score of the operation [8], CPB duration (minutes), priming volume (mL), lowest temperature (°C) reached while on CPB, and lowest hematocrit (%) reached while on CPB.

The duration of storage time of the RBCs used during and after CPB was obtained from our computerized blood bank files. Records of metabolic data during CPB were collected from perfusionists' files.

After 10 minutes on CPB, the following data were collected: arterial pH, arterial pCO_2 _(mmHg), and arterial base excess as well as potassium (mEq/L), calcium (mEq/l), lactate (mmol/L), and glucose (mg/dL) blood concentrations. The peak values of potassium, lactate and glucose obtained during CPB were also collected.

Outcome variables were derived from our institutional database. The following variables were collected: mechanical ventilation time (hours), intensive care unit (ICU) stay (hours), blood loss (mL/12 hours), peak postoperative creatinine level (mg/dL), and peak postoperative bilirubin level (mg/dL) as well as the need for postoperative allogeneic RBC, fresh frozen plasma, or platelet transfusions. Postoperative morbidity and mortality data were also collected. Parameters collected included data regarding low cardiac output (defined as the need for inotropic support for more than 48 hours postoperatively), acute renal failure (ARF) (defined as the need for renal replacement therapy), pulmonary complications (defined as respiratory distress syndrome or pneumonia), neurological complications (defined as stroke, coma, or neurologic defects still present at hospital discharge), gastroenterological complications (defined as bleeding, necrotizing enterocolitis, or liver failure), sepsis, and in-hospital mortality.

Major morbidity was defined as the presence of one or more of the following: ARF, sepsis, or pulmonary, neurological, or gastroenterological complications.

### Group definitions

The patients were divided into two groups: patients receiving newer blood and patients receiving older blood. The storage time of the RBCs used was analyzed using the following steps:

1. For patients receiving more than one unit of RBCs, the oldest unit of RBCs received was used for group allocation.

2. The median value of blood storage time was assessed, and patients were attributed to the *newer blood *group if they received only blood that had been stored for a period of time (days) equal to or shorter than the median value. Patients were attributed to the *older blood *group if they received any amount of RBC stored for a period of time longer than the median value.

3. For analysis of the metabolic data during CPB, the same procedure was followed, but only the unit of blood used for priming the circuit was taken into consideration when allocating patients to groups based on the duration of RBC storage.

4. The group of patients receiving only postoperative transfusions was selected based on an age range similar to the blood-prime group.

### Statistics

Continuous variables are presented as median values and interquartile ranges, and categorical variables are presented as numbers and/or percentages in the tables and the text. To compare data between groups, we used two-sided tests. The Wilcoxon rank-sum test was used to compare continuous variables and Pearson's chi-square test was used to compare categorical variables. Yates correction was applied when appropriate.

In order to better elucidate the relationship between RBC storage time and the primary endpoint variable (major morbidity), we performed a logistic regression analysis based on the oldest RBCs each patient received. To adjust for potential confounders, other pre- and intraoperative factors thought to be associated with conditions included in the definition of major morbidity were explored. A forward stepwise multivariable logistic regression analysis was performed to detect whether or not RBC storage time was an independent risk factor for major morbidity. The same analysis was applied to relevant single morbidity events.

## Results

### Blood-prime group

#### Age of RBCs used in transfusions

The median storage time of the RBCs used for priming the CPB machine and for subsequent intraoperative transfusions was four days (range: 1 to 18 days). The storage time of the RBCs used only for priming the CPB machine had the same range of values and median value. Therefore, a four-day cut-point was used to divide patients into groups. The patients were allocated to groups according to the storage time of the oldest blood they received either to the newer blood group (one to four days of storage time, N = 116) or the older blood group (more than four days of storage time, N = 76). For the purposes of the analysis of metabolic changes during CPB, only the storage time of the blood used for priming the CPB machine was considered, and the composition of the two groups therefore differed slightly (newer blood: N = 123; older blood: N = 69).

All patients were exposed to at least one unit of RBCs, which was used to prime the CPB machine. One hundred eighty-seven patients (97.4%) were exposed to a second unit of RBC, which was used for intraoperative transfusions during or after CPB.

Table 1 lists the demographic features, preoperative characteristics, and intraoperative data for patients receiving newer or older blood. The ages range from two days to five years. The two groups did not differ in terms of the total number of units of RBCs to which they were exposed or in terms of the total volume of RBCs they were given. Patients in the older blood group received RBCs that had been stored for a median of six days, which was significantly longer than patients in the newer blood group, who received blood that had been stored for a median of three days. Patients receiving older blood had a significantly higher preoperative hematocrit than patients receiving newer blood. No other differences were found between the two groups.

**Table 1:** Demographic information, preoperative profile and operative details in the blood-prime group

Variable	Patients Receiving Newer Blood (N = 116)	Patients Receiving Older Blood (N = 76)	*P *value
**Transfused blood**			
Duration of storage (days)			
Median	3	6	0.001
Interquartile range	2 to 4	5 to 8	
Number of red blood cell units			
Median	2	2	0.985
Interquartile range	2 to 2	2 to 2	
**Demographic features**			
Age (months)			
Median	6.5	8	0.089
Interquartile range	4 to 11	5 to 11	
Male sex -- no. of patients (%)	68 (58)	37 (49)	0.196
Weight (kgs)			
Median	6.15	6.7	0.244
Interquartile range	4. 5 to 7.6	5 to 7.7	
**Clinical features**			
Hematocrit (%)			
Median	33	36	0.005
Interquartile range	31 to 36	32 to 38	
Platelet count (cells/μL)			
Median	321000	314000	0.903
Interquartile range	249000 to 382000	245000 to 391000	
Prothrombin activity (%)			
Median	84.5	84.7	0.903
Interquartile range	76 to 91	76 to 94	
aPTT (seconds)			
Median	31.2	31	0.724
Interquartile range	28 to 34	29 to 34	
Antithrombin activity (%)			
Median	105	104	0.190
Interquartile range	91 to 115	89 to 113	
Creatinine (mg/dL)			
Median	0.3	0.3	0.429
Interquartile range	0.25 to 0.35	0.25 to 0.35	
Bilirubin (mg/dL)			
Median	0.4	0.35	0.509
Interquartile range	0.27 to 0.65	0.27 to 0.54	
Aristotle score			
Median	7.5	6.3	0.803
Interquartile range	6 to 8	6 to 8	
Redo operations -- no. of patients (%)	6 (5.2)	4 (5.1)	0.296
**Operative details**			
Procedure -- no. of patients (%)			
ASD repair	2 (2)	2 (3)	
VSD repair	39 (34)	27 (36)	
TOF correction	18 (16)	12 (16)	
Arterial switch	10 (9)	10 (13)	
AV canal	16 (14)	10 (13)	
Others	31 (25)	16 (19)	
Priming volume (mL)			
Median	400	400	0.292
Interquartile range	380 to 400	380 to 400	
Transfused RBC volume (mL)			
Median	240	240	0.792
Interquartile range	216 to 300	240 to 300	
CPB duration (minutes)			
Median	82	74	0.480
Interquartile range	58 to 110	52 to 120	
Lowest temperature (°C)			
Median	29.6	30	0.771
Interquartile range	28.3 to 31	28 to 32	
Lowest hematocrit (%)			
Median	28	28	0.151
Interquartile range	26 to 29	26 to 30	

aPTT = activated partial thromboplastin time; ASD = atrial septal defect; AV = atrioventricular; CPB = cardiopulmonary bypass; TOF = tetralogy of Fallot; VSD = ventricular septal defect.

Continuous variables are described as medians and interquartile ranges, and *P *values were calculated by the Wilcoxon rank-sum test; categorical variables are described as numbers and percentages, and *P *values were calculated by Pearson's chi-square test.

#### Metabolic data during CPB

The metabolic profiles obtained from patients while on CPB are reported in Table 2. There were no significant differences noted between the two groups in terms of values obtained after 10 minutes of CPB or peak values obtained during CPB.

**Table 2:** Acid-base balance, electrolyte, lactate, and glucose levels during cardiopulmonary bypass (after 10 minutes on CPB and peak levels recorded during CPB)

Variable	Patients Receiving Newer Blood (N = 123)	Patients Receiving Older Blood (N = 69)	*P *value
pH			
Median	7.41	7.42	0.432
Interquartile range	7.36 to 7.44	7.37 to 7.47	
pCO2 (mmHg)			
Median	42	40	0.297
Interquartile range	36 to 46	35 to 43	
Base excess			
Median	1.4	1.3	0.994
Interquartile range	-1.2 to 4.3	-1.3 to 3.8	
Potassium (mEq/L)			
Median	4.7	4.6	0.387
Interquartile range	3.7 to 5.6	3.5 to 5.3	
Calcium (mEq/L)			
Median	1.4	1.4	0.168
Interquartile range	1.2 to 1.6	1.2 to 1.6	
Lactate (mmol/L)			
Median	1.8	1.9	0.127
Interquartile range	1.2 to 2.3	1.5 to 2.5	
Glucose (mg/dL)			
Median	142	141	0.469
Interquartile range	127 to 168	121 to 165	
Peak potassium (mEq/L)			
Median	5.3	5.6	0.746
Interquartile range	4.7 to 6	4.8 to 6	
Peak lactate (mmol/L)			
Median	1.9	2.0	0.237
Interquartile range	1.5 to 2.5	1.5 to 2.8	
Peak glucose (mg/dL)			
Median	175	186	0.630
Interquartile range	154 to 215	143 to 227	

Variables are described as medians and interquartile ranges, and *P *values were calculated by the Wilcoxon rank-sum test.

#### Clinical outcomes

Fifteen patients (12.9%) experienced at least one morbidity event in the newer blood group, and 18 (23.7%) in the older blood group (*P *= 0.053).

**Figure 1:**
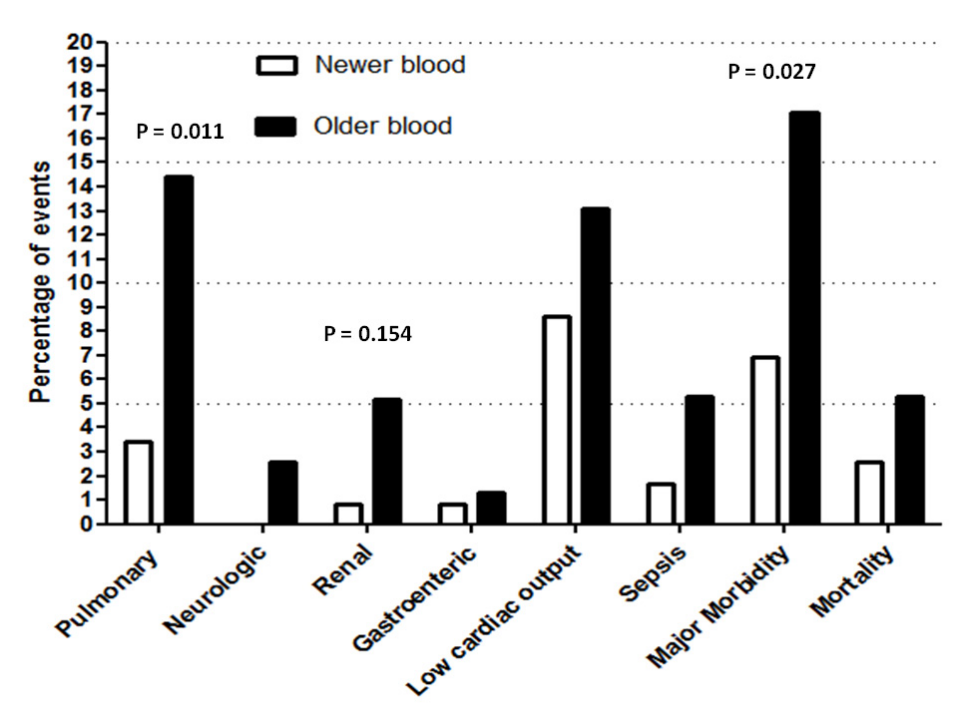
Morbidity and mortality in patients receiving newer vs. older blood in the cardiopulmonary bypass circuit.

Patients receiving older blood had a significantly higher rate of certain complications (Figure 1). Major morbidity was found to be present in 17.1% of the patients receiving older blood vs. 6.9% in patients receiving newer blood (*P *= 0.027). The pulmonary complication rate was significantly higher in patients receiving older blood as compared to patients receiving newer blood (14.4% vs. 3.5%; *P *= 0.011). The ARF rate was 5.2% in patients receiving older blood and 0.8% in patients receiving newer blood (*P *= 0.154), and the rate of infectious complications was 5.5% in patients receiving older blood and 1.7% in patients receiving newer blood (*P *= 0.223).

Other outcome data are reported in Table 3. Patients receiving older blood had a significantly higher rate of platelet transfusions, while the other parameters did not differ significantly between groups.

**Table 3:** Outcome data for groups receiving newer vs. older blood in the blood-prime group

Variable	Patients Receiving Newer Blood (N = 116)	Patients Receiving Older Blood (N = 76)	*P *value
Mechanical ventilation time (hours)			
Median	48	48	0.638
Interquartile range	13 to 109	11 to 120	
ICU stay (hours)			
Median	96	96	0.702
Interquartile range	48 to 150	48 to 192	
Peak creatinine (mg/dL)			
Median	0.55	0.60	0.334
Interquartile range	0.40 to 0.90	0.40 to 0.80	
Peak bilirubin (mg/dL)			
Median	1.3	1.4	0.503
Interquartile range	0.62 to 2.12	0.79 to 2.00	
Blood loss (mL/12 hours)			
Median	70	60	0.156
Interquartile range	40 to 100	30 to 100	
Red blood cells transfusions			
No. of patients (%)	69 (59)	47 (62)	0.760
Fresh frozen plasma transfusions			
No. of patients (%)	55 (47)	39 (51)	0.607
Platelets transfusions			
No. of patients (%)	4 (3.5)	9 (12)	0.024

ICU = intensive care unit.

Continuous variables are summarized by medians and interquartile ranges, and *P *values were calculated by the Wilcoxon rank-sum test; categorical variables are summarized by numbers and percentages, and *P *values were calculated by the Pearson's chi-square test.

**Figure 2:**
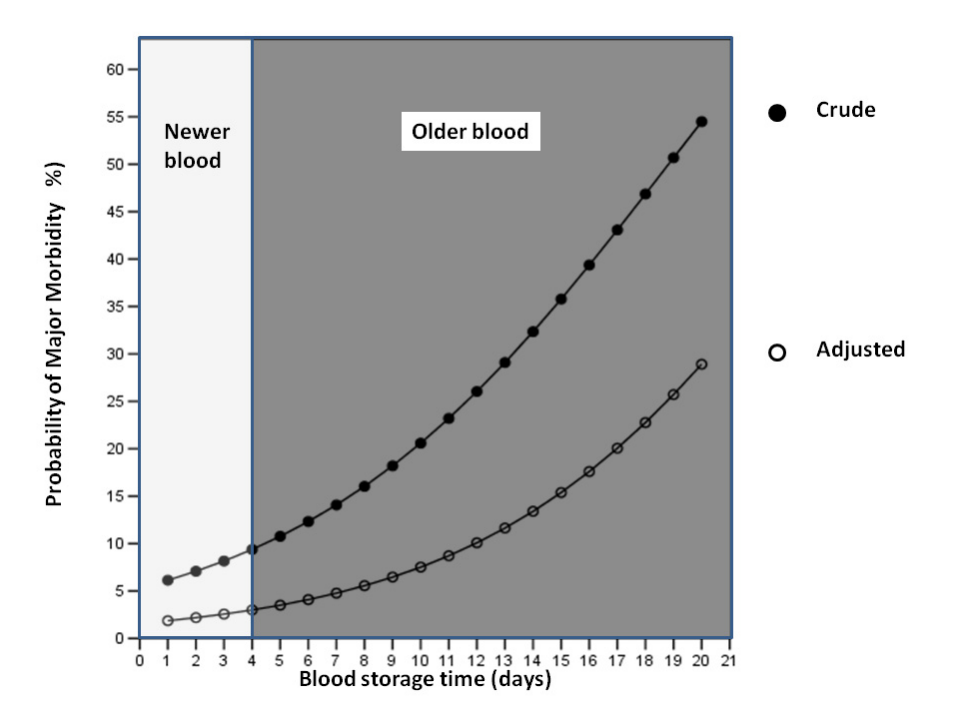
Crude and adjusted likelihood of experiencing major morbidity in the group receiving blood prime.

The association between RBC storage time and the primary endpoint variable (major morbidity) was explored using a sensitivity analysis, in which RBC storage time was treated as a continuous variable. On univariate logistic regression analysis, RBC storage time was found to be significantly associated with major morbidity (*P *= 0.016). Other pre- and intraoperative variables were explored for a possible association with major morbidity. Factors found to be significantly associated with major morbidity were preoperative hematocrit, the preoperative serum creatinine value, the Aristotle score, and CPB duration. These factors were entered into a multivariable logistic regression analysis along with the RBC storage time. Through a forward stepwise process, a final multivariate model was created in which only CPB duration and RBC storage time remained independent predictors of major morbidity (Table 4). Both the unadjusted and adjusted models reporting the likelihood of major morbidity as a function of the RBC storage time are included in Figure 2.

**Table 4:** Multivariable logistic regression analysis for risk of major morbidity in the blood-prime group

Variable	B	SE	OR	95% CI	*P *value
CPB duration (minutes)	0.011	0.004	1.011	1.004 to 1.018	0.003
Blood storage time (days)	0.161	0.069	1.175	1.026 to 1.345	0.020
Constant	-4.122				

b = regression coefficient; C.I. = confidence interval; CPB = cardiopulmonary bypass; OR = odds ratio; SE = standard error

The association between RBC storage time and pulmonary complications was addressed with the same analysis. Since it is known that platelet transfusions may trigger pulmonary complications due to the presence of plasma and the intra-pulmonary accumulation, platelets were included in the multivariable model. Again, the only independent factors for pulmonary complications were RBC storage time and CPB duration (Table 5). There was a non-significant (*P *= 0.382) trend for association between platelet transfusions and pulmonary complications.

**Table 5:** Multivariable logistic regression analysis for risk of pulmonary complications in the blood-prime group

Variable	B	SE	OR	95% CI	*P *value
CPB duration (minutes)	0.012	0.004	1.012	1.004 to 1.020	0.003
Blood storage time (days)	0.197	0.079	1.218	1.043 to 1.423	0.013
Platelet transfusion	0.830	0.848	2.293	0.435 to 12.09	0.328
Constant	-4.9				

b = regression coefficient; C.I. = confidence interval; CPB = cardiopulmonary bypass; OR = odds ratio; SE = standard error.

### Post-CPB transfusions group

Ninety-eight patients received only post-CPB RBC transfusions. This group was highly non-homogeneous with respect to the blood-prime group. They were significantly (*P *< 0.001) older (46 ± 37 months vs. 9 ± 9 months), with a higher weight (13 ± 7 kg vs. 6.5 ± 2.7 kg) and baseline hematocrit (40 ± 7.6% vs 34 ± 4.6%). To adjust for age differences, we included in this group only patients aged five years or less (same range of the blood-prime group), sorting out 47 patients. Despite this, this group remained composed by significantly (*P *< 0.001) older (38 ± 17 months) patients with higher weight (13 ± 4 kg) and hematocrit (40.8 ± 7.8%). Therefore, these data were not pooled together with the blood-prime group, but analyzed separately.

The median RBC storage time was four days (range 1 to 26, interquartile range 3 to 8). Twenty-five patients received newer blood and 22 older blood. This group experienced fewer complications than the blood-prime group, with only 4.3% of major morbidity, 6.5% of low cardiac output, 2.1% of pulmonary complications, 2.1% of sepsis and no mortality. No association was found between RBC storage time and any complication.

## Discussion

Intraoperative transfusion of RBC that had been stored for more than four days was associated with a significantly increased risk of postoperative complications in newborns, infants, and children aged five years or less undergoing cardiac surgery when blood was used to prime the CPB circuit. The pulmonary complication rate was significantly higher in patients receiving older blood, and a higher major morbidity rate (a measure of serious complications) was observed in patients receiving older blood.

After adjusting for other explanatory variables, there was a significant association found between RBC storage time and the risk of major morbidity. The storage time of RBC used for priming the CPB circuit was not associated with metabolic changes immediately after the onset of CPB or during the entire course of CPB.

There is little information available in the literature about the impact of RBC storage time on the intra- and postoperative courses of pediatric patients undergoing CPB. Several articles [9,10] have explored the metabolic effects of using fresh vs. older stored blood in CPB priming solution, but these studies have limited their analysis to effects observed during CPB. Another study [11] compared blood lactate levels and clinical outcomes in pediatric patients treated with either blood-containing or bloodless priming solutions.

There is conflicting information in the literature with respect to metabolic changes that occur during CPB with respect to the use of fresh or old blood. Schroeder and coworkers [10] found that pediatric patients receiving RBC stored for more than 12 days had higher blood lactate levels and lower blood glucose levels during CPB in comparison to patients receiving RBC stored for 12 days or less. They identified a linear association between RBC storage time and both blood lactate and glucose levels during CPB. However, at the end of the operation, no differences in blood lactate levels were detected. Conversely, Keidan and coworkers [9] did not find any metabolic difference in blood electrolytes, lactate, or glucose levels during CPB in patients receiving newer (storage time less than or equal to five days) vs. older (storage time more than or equal to five days) blood in the CPB priming solution.

Our study is in agreement with the results of Keidan and coworkers but uses a model that considers both the metabolic values after the onset of CPB and the peak values obtained during CPB. It is possible that the different cut-off values used in various studies (we used cut-offs that were similar to those used by Keidan and coworkers, but the cut-offs used in Schroeder's study were much longer) may explain these different results. However, a common finding in all of these studies is that the blood lactate level at the end of the operation or upon arrival in the ICU was not associated with the storage time of the blood used in the priming solution. Moreover, there is evidence that blood lactate levels during the ICU stay are initially higher in pediatric patients receiving a bloodless priming solution than in those receiving a blood-containing priming solution [11]. However, the association between early postoperative blood lactate levels and outcomes following pediatric cardiac operations is still not well defined [11-13].

The present study essentially confirms, in a population of newborns, infants and children aged five years or less and receiving cardiac operations with blood prime, the finding of Koch and coworkers [4] that RBC storage time is an independent predictor of morbidity. However, there are some major differences between the two studies (apart from a different sized patient population).

### (i) Cut-off values for the RBC storage time

In Koch's study, a cut-off of 15 or more days was used to define the older blood group. This was based on previous observations that functional and structural changes of stored RBCs begin after two to three weeks of storage [14,15]. Incidentally, this value was close to the median value for their patient population, and the two groups were similar in numbers. In our study, we used the median value of the RBC storage time as the cut-off point for dividing the two groups. Our cut-off time is considerably shorter than that used by Koch et al. but is similar to cut-offs used in other pediatric studies [9]. This is due to the generally accepted clinical practice of preferentially using fresh RBCs for priming CPB circuits in this patient population. It is our feeling that searching for a universal cut-off value for RBC storage time is useful for statistical purposes, but somewhat arbitrary when addressing the influence of RBC storage time on postoperative outcomes. Both in our study and in the study by Koch et al. [4], RBC storage time was identified as an independent risk factor for morbidity even when examined as a continuous variable. The risk of experiencing an outcome included in the major morbidity definition was found to be increased even for increased storage times below the cut-off value. In our pediatric study, the risk of experiencing one of these outcomes among patients receiving RBCs stored for four days is about twice as high as that for patients receiving RBCs stored for one day. In the adult study [4], the risk of experiencing one of the outcomes included in the definition of composite morbidity was about 50% higher for patients receiving RBCs stored for 14 days than for patients receiving RBCs stored for one day.

### (ii) Time-event relationship between transfusions and outcome

In our study, we could find a significant association between RBC storage time and outcome only when examining the effect of RBCs used to prime the CPB circuit. Conversely, in Koch's study, they included RBC transfusions that occurred throughout the entire hospital stay. We could not include patients receiving only post-CPB RBCs transfusions in the blood-prime group, because due to the standard practice in cardiac surgery, patients receiving *clear prime *are greatly different for age, weight, and baseline hematocrit if compared with patients treated with blood prime. However, in a sub-analysis of patients receiving only post-CPB transfusions we could not find any association between RBC storage time and outcome. There are different explanations for this finding. The most likely is that due to the limited number of patients in this group, and the very few adverse events, we were lacking the power to detect differences. Moreover, we can be sure that the events included in the major morbidity in the blood-prime group always occurred following RBC transfusion. Conversely, it is possible that the complication had occurred prior to the transfusion (and thus been a possible reason for the transfusion), in patients receiving RBCs only after CPB. This could explain the lack of association between the two events in this second group.

Finally, it is likely that old blood may be more susceptible to the mechanical insult of CPB than new blood, and this effect is of course lacking in patients not receiving blood prime.

In pediatric patients as well as in adult patients, the pathophysiological mechanisms underlying the association between RBC storage time and adverse outcomes remains unclear. The *storage lesion *that occurs in the RBC is a combination of physical and biochemical changes, which include the depletion of 2,3-diphosphoglycerate [16], the formation of proinflammatory cytokines [17], a decrease in RBC deformability [14], and an increase in RBC adhesiveness and aggregability [17]. These last two factors may contribute to impaired microvascular blood flow [14,17,18], which may affect organ function, especially in the organs acting as natural filters, such as the lung and the kidney.

It is notable that in our study, pulmonary complications were observed significantly more frequently in patients receiving older blood. Additionally, the rate of ARF was observed to be higher in the same group and this difference was very close to reaching statistical significance. These two complications were the most important contributors to the significantly higher major morbidity rate observed in patients receiving older blood. Systemic infections occurred at a non-significantly higher rate in patients receiving older blood. This could be because the leukocytes present in the RBC units might have had deleterious effects on the immune system, provoking a state of immunosuppression and making individuals more susceptible to postoperative infections. The bioactive substances released by leukocytes may be responsible for this effect. Since there is a time-dependent accumulation of these substances in blood components during storage, individuals receiving blood stored for a longer period of time may have a higher susceptibility to infection than individuals receiving blood stored for a shorter period of time. An association between RBC storage time and infection rates has been found by other authors in adult patient populations [4,5,19].

### Limitations of this study

The main limitation of this study is the relatively small study population. However, studies addressing outcomes after cardiac operations in pediatric patients aged five years or less cannot include as many patients population as studies focused on adult patients because the overall population size is smaller. The population size of the present study was large enough to explore morbidity, but not mortality. The fact that even with a limited number of patients and events we could identify significant associations between morbidity outcome variables and RBC storage time is probably due to the large impact that one or two units of stored RBCs may have in patients with a small body surface area. Even one or two units of RBCs can be considered a massive transfusion in very small pediatric patients.

A second limitation of the study is related to the impossibility of exploring the role of leukodepleting techniques with respect to the effects of RBC transfusions in this retrospective patient sample. All our patients received non-leukodepleted blood. However, the role of leukodepletion in limiting the adverse effects of RBC transfusions is still unclear and a benefit has not been demonstrated in pediatric patients undergoing cardiac surgery. Additionally, in Koch's study, patients receiving older blood had significantly worse outcomes despite receiving leukodepleted blood at a significantly higher rate than patients receiving newer blood.

## Conclusions

The storage time of RBCs used for priming the CPB circuit during cardiac operations is associated with increased major morbidity and primarily affects pulmonary and renal function. There is a significant association between the storage time of RBCs and the risk of experiencing a serious postoperative complication. This risk increases with increasing storage time even within the group of patients receiving newer (less than five-day-old) blood. Therefore, the use of the freshest possible blood is suggested for priming the CPB circuit. We could not identify the same association between RB storage time and bad outcomes in patients being transfused but not receiving blood prime. A further study including a larger patient population is needed to address this point.

## Key messages

• In a population of pediatric patients including newborns, infants, and children aged five years or less undergoing cardiac operations with CPB and receiving RBCs to prime the CPB circuit the RBC storage time is associated with an increased morbidity rate.

• Patients receiving older (more than four days storage time) blood had a higher rate of pulmonary complications and major morbidity.

• After correction for confounding factors, RBC storage time remains independently associated with pulmonary complications and major morbidity.

• This association was not found in a population of patients of the same age not receiving blood prime but being transfused after CPB.

## Abbreviations

aPTT: activated partial thromboplastin time; ARF: acute renal failure; ASD: atrial septal defect; AV: atrioventricular, CPB: cardiopulmonary bypass; ICU: intensive care unit; RBC: red blood cells; TOF: tetralogy of Fallot; VSD: ventricular septal defect.

## Competing interests

The authors declare that they have no competing interests.

## Authors' contributions

MR was involved in study design, statistical analysis, manuscript preparation. CC was involved in data acquisition and interpretation. GI was involved in data acquisition and interpretation, and manuscript drafting. SB and AB were involved in data acquisition. AF was involved in data interpretation and manuscript drafting. TDLT was involved in statistical analysis. DDB did manuscript preparation and study design.
